# THE PREVALENCE AND CHARACTERISTICS OF UNREPRESENTED ADULTS ADMITTED TO KOREAN HOSPITAL SETTINGS

**DOI:** 10.1093/geroni/igad104.3089

**Published:** 2023-12-21

**Authors:** Hyejin Kim, Jiyeon Choi, Won Lee, Ilhak Lee, Mi-Kyung Song

**Affiliations:** Chung-Ang University, Seoul, Republic of Korea; Yonsei University, Seoul, Republic of Korea; Chung-Ang University, Seoul, Republic of Korea; Yonsei University, Seoul, Republic of Korea; Emory University, Atlanta, Georgia, United States

## Abstract

**BACKGROUND:**

Unrepresented adults refer to persons who have lost their decision-making capacity and have neither available surrogates to make medical decisions for them nor the documentation of their treatment preferences. Recently, concerns about this population have been emerging in the hospital setting in South Korea. However, little is known about this population. Thus, this study aimed to identify the prevalence and demographic and clinical characteristics of unrepresented patients admitted to hospitals in South Korea.

**METHODS:**

This qualitative descriptive study was conducted using semi-structured interviews with 37 physicians, nurses, social workers, and others from 22 hospitals and directed content analysis. RESULTS: The number of unrepresented patients seemed to be small in hospital settings, but higher in public hospitals than private ones. Participants reported that the reasons unrepresented patients lost their decision-making capacity included loss of consciousness, delirium or alcoholism-related mental change, dementia, and mental retardation. Surrogates were unavailable because no families were found through a rigorous search, or because families were inaccessible, refused to be decision-makers, or were unable to serve as surrogates as they were not a legally-bound immediate family. These patients were mostly in their 50s – 70s, predominantly male, and living alone, and had poor socioeconomic status and poor hygiene. Most of them had no medical records before admission. CONCLUSIONS: Unrepresented patients are recognizable even though the number is small. Based on the common characteristics of unrepresented adults, national and societal efforts are necessary to identify ‘at-risk’ people and prevent them from becoming unrepresented in healthcare settings.

